# Tinea Incognito—A Great Physician Pitfall

**DOI:** 10.3390/jof8030312

**Published:** 2022-03-18

**Authors:** Julia Nowowiejska, Anna Baran, Iwona Flisiak

**Affiliations:** Department of Dermatology and Venereology, Medical University of Bialystok, Zurawia 14 St., 15-540 Bialystok, Poland; anna.baran@umb.edu.pl (A.B.); iwona.flisiak@umb.edu.pl (I.F.)

**Keywords:** tinea incognito, tinea, dermatophytes, glucocorticoids, terbinafine, itraconazole, fungal infection

## Abstract

Tinea incognito is a dermatophyte infection exacerbated after inadequate administration of topical or systemic glucocorticoids. A 57-year-old man presented to the Department of Dermatology due to skin lesions persisting for one month. He reported having recently worked under hot conditions, in tight clothing, which caused sweating. Later, he noticed erythematous–exfoliative lesions in his groins and on the buttocks. He presented to the general practitioner who diagnosed him with eczema and prescribed clobetasole ointment. Since the skin lesions became more severe, he presented to the Department of Dermatology. On the physical examination, extensive erythematous–infiltrative lesions were observed in the area of medial, lateral, and posterior surface of both thighs and buttocks. Pustules were also present. Suspicion of tinea incognito was raised, and direct mycological examination and culture confirmed the presence of dermatophytes. The patient was prescribed topical terbinafine and oral itraconazole. Tinea incognito may be challenging to diagnose because the clinical presentation is relatively nonspecific and definitive culture or histopathological diagnosis such as by microscopic sample examination to identify fungal elements is not universally available. Every doctor has to keep in mind the fact that tinea may be a great mimicker of other dermatoses and to not prescribe medications without microscopic confirmation of tinea, and refer patients for dermatological consultation in case of doubt.

## 1. Introduction

Tinea corporis is a superficial fungal skin infection caused by dermatophytes, of which *Trichophyton rubrum* seems to be the most common [1]. It involves glabrous skin of the trunk and extremities, excluding palms, soles, and folds [2,3]. Typical clinical presentation involves erythematous patches or plaques of circular or ovoid shape, sharply demarcated with a raised edge [3]. Exfoliation may vary in degree [3]. Vesicles and pustules may also be present at the border of the lesions [3,4]. It may be accompanied by subjective symptoms such as pruritus or burning sensations [3]. The risk factors for tinea development are familial and genetic predisposition (due to specific defects in innate or adaptive immunity, e.g., low defensin β4), diabetes mellitus, lymphomas, immunodeficiency, Cushing’s syndrome, excess sweating, or older age [3,5]. The diagnosis of tinea corporis is mainly established clinically and is often confirmed by direct microscopy and fungal culture of skin scrapings. [2]. Tinea may be a great diagnostic challenge since it may mimic many other dermatoses, and several other skin diseases may present similarly to tinea. The treatment consists of topical and/or systemic antifungal agents, of which the most commonly used are terbinafine and azoles, and especially itraconazole [3].

Tinea incognito is a dermatophyte infection exacerbated after inadequate administration of glucocorticoids, topical or systemic [6]. Some authors suggest that the application of topical calcineurin inhibitors (tacrolimus, pimecrolimus) may also be the reason for such atypical tinea [7], but the clinical presentation seems to be similar to the one after glucocorticoids administration [8]. Tinea incognito was first described in 1968 [9], but the literature data suggest that the incidence has increased in recent years [4]; moreover, doctors of every specialty may encounter this problem, so it is important to report on such cases and suggest how to avoid them.

We present a case report of a patient who developed classic tinea corporis, which was unfortunately misdiagnosed and treated with topical glucocorticoids, which lead to extensive tinea incognito.

## 2. Case Description

**Figure 1:**
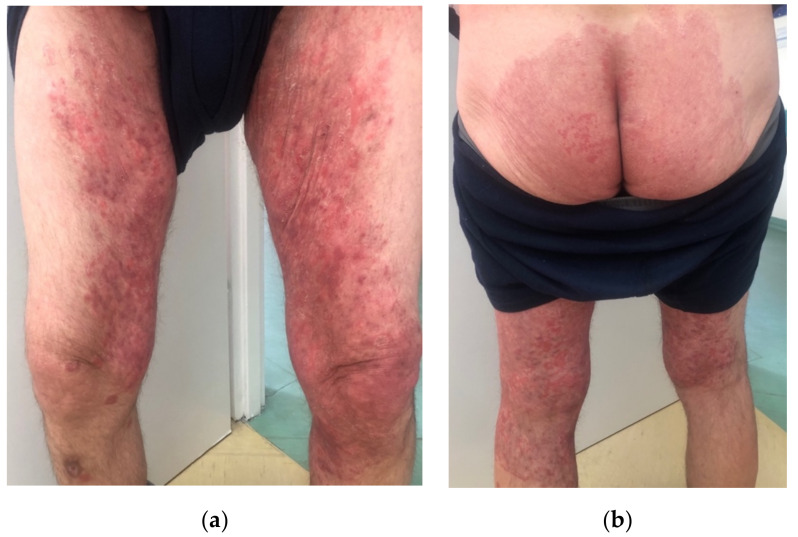
At admission. Erythematous–infiltrative lesions with pustules in the medial and posterior area of both thighs and groins (**a**), erythematous–exfoliative lesions on the buttocks (**b**).

**Figure 2:**
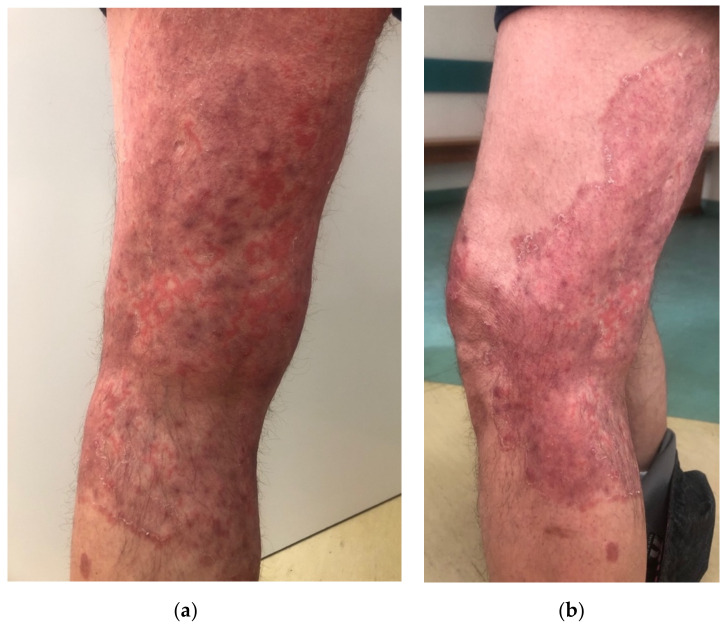
At the admission. Close-up view of the lesions on of the left tight, posterior (**a**) and lateral (**b**) side.

A 57-year-old male, with a history of type 1 diabetes mellitus (complicated by polineuropathy, treated with insulin) and arterial hypertension (treated with perindopril), presented to the Department of Dermatology due to skin lesions persisting for one month. He was a manual worker and reported that he recently worked outside during hot weather in June, in tight, unbreathable-material clothing, which caused intensive sweating. After some time, he noticed erythematous–exfoliative lesions in his groins and on the buttocks, accompanied by pruritus. He presented to a general practitioner, who diagnosed him with eczema and prescribed clobetasole ointment. The patient administered the ointment once or twice a day for a month with no improvement, but deterioration. Moreover, he topically applied mupirocin, and gentamicin with bethametasone and took amoxicillin with clavulanic acid orally. Since the skin lesions became more severe and extensive, he presented to the Department of Dermatology. On the physical examination, extensive erythematous–infiltrative lesions were observed in the area of medial, lateral, and posterior surface of both thighs and buttocks, with satellite lesions visible (Figure 1a,b and Figure 2a,b). Pustules were also present in some areas.

**Figure 3:**
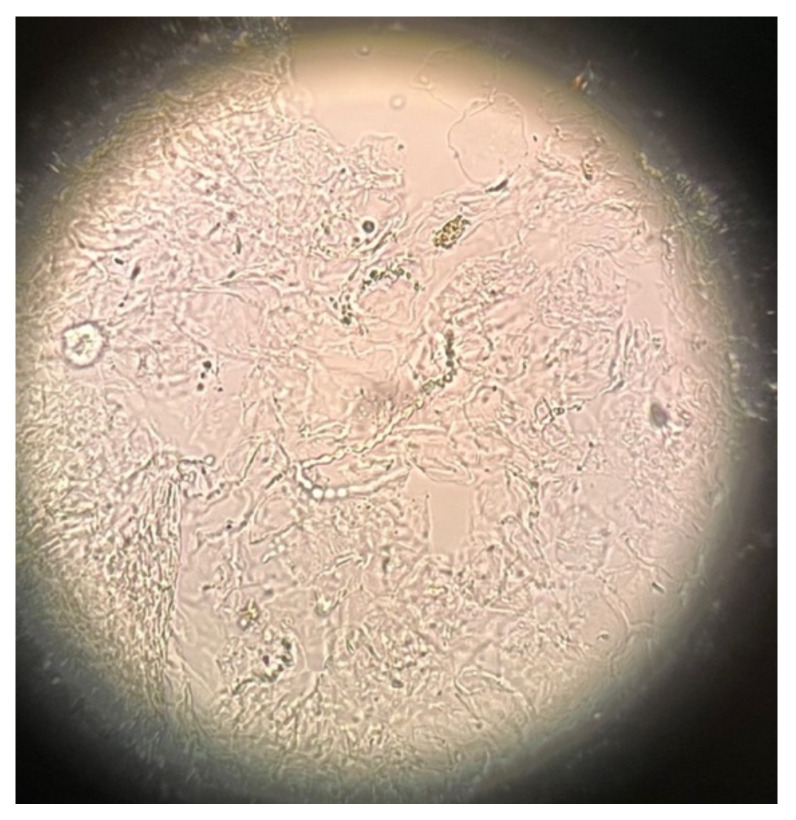
Microscopic mycological examination of the skin scrapings (20× magnification). Long narrow hyphae.

The dermatologist raised the suspicion of tinea incognito and referred the patient instantly for the direct mycological examination, which revealed long narrow hyphae, most probably dermatophytes (Figure 3). The culture had also been started. The patient was strongly forbidden to use glucocorticoids and was prescribed topical terbinafine cream and oral itraconazole at a dose of 200 mg twice a day.

**Figure 4:**
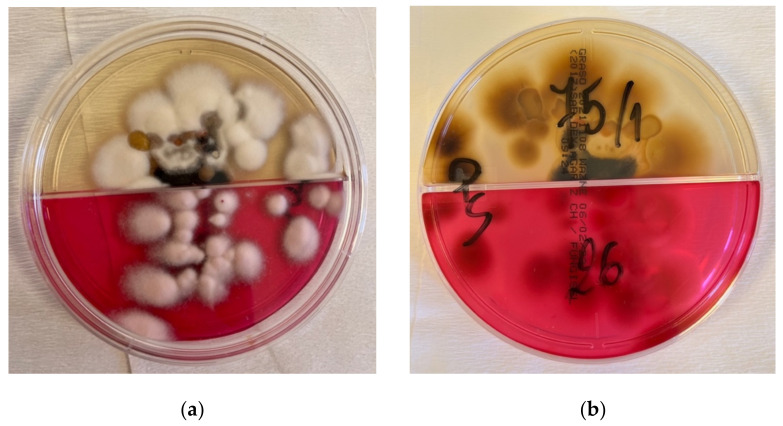
Culture grown from the skin lesions scrapings. Cottony, powdery, cream-beige colonies of *Trichophyton mentagrophytes* (**a**), with brown pigmentation on the back (**b**).

The patient came back for the follow-up a week later and there was already a visible improvement. The lesions became more pale and less inflammatory. However, the doctor noticed yellowish coloration of his sclerae. He was therefore advised to perform several laboratory tests: aminotransferases and gamma-glutamyl transferase activity, as well as bilirubin concentration. The latter turned out to be elevated. The patient also made an impression of an alcohol abuse habit, which he strongly denied. He was advised to lower the dose of oral itraconazole to 100 mg per day and discouraged to drink any alcohol drinks. After 4 weeks, he presented to the ambulatory care for a follow-up; a great improvement in skin lesions was observed, and the results of laboratory tests were within normal limits. The culture grown from the skin lesions scrapings revealed *Trichophyton mentagrophytes* (Figure 4a,b). The treatment was discontinued, and the patient was given instructions on personal hygiene, including work hygiene.

**Figure 5:**
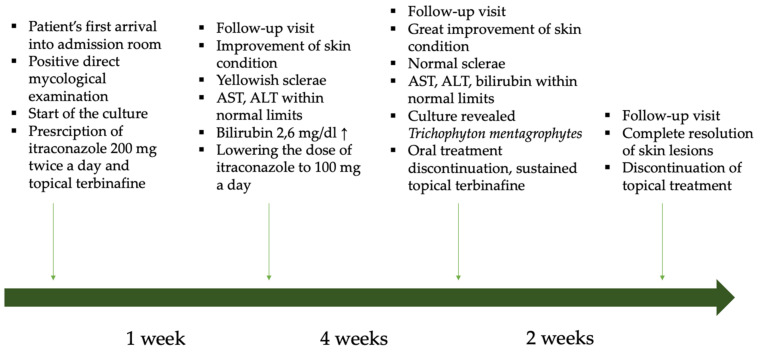
The diagram illustrating the whole patient’s history.

The whole patient’s history is presented in the diagram in Figure 5.

## 3. Discussion

Tinea incognito, which is an exacerbated manifestation of dermatophytosis, may present differently from regular tinea. Its pathogenesis is explained by the altered-by-steroids response of the host to cutaneous fungal infection [10]. Based on a literature review, the most common causative factor of tinea incognito seems to be *Trichophyton rubrum*, followed by *Trichophyton mentagrophytes* and *Epidermophyton floccosum* [11,12,13], whereas the most common location seems to be the limbs [11] or trunk [13]. Tinea incognito can manifest as eczema-like, lichenoid, rosacea-like, and psoriasis-like lesions, sometimes even bullous, of which the first one seems to be the most frequent [11,13,14,15]. In the case of our patient, it could mimic eczema; therefore, the patient was probably prescribed glucocorticoids. The most common conditions that could be mistaken with tinea according to the available literature data, depending on the particular location, are presented in Table S1 in the Supplementary Materials [11,12,16]. Tinea incognito seems to appear with similar frequency in every age group, besides infancy and the elderly aged above 75 years old [17].

Two issues are essential when discussing tinea incognito. The first one is its prevention, and the second is its diagnosis and treatment.

Tinea corporis diagnosis is usually made based on the patient’s history and physical examination, which can already raise the suspicion of fungal infection. A very important part of the diagnostic process is the mycological examination, which may quickly confirm the suspicion of tinea and point to the adequate treatment. A great problem, especially in our, and presumably also other countries, is the lack of access to direct mycological examination in the general practice. The only solution is to advise the patient to have the test performed in an external laboratory for a fee, which discourages both doctors and patients. Family doctors are so-called ‘gate keepers’, and they are usually the first people to see the patient. As they cannot perform mycological examination, since it is not available in their pool of services, they may easily misdiagnose tinea. It is a great mimicker and can often be mistaken with eczema, psoriasis, lupus erythematosus, or seborrhoeic dermatitis [18]. Hence, glucocorticoids are often inadequately prescribed, which leads to the development of tinea incognito. A similar situation may occur when the patient does not present to the doctor and tries to treat the lesions on his own. Several topical glucocorticoids or their combinations with other substances are available in many countries over-the-counter, which enables easy access to such agents and may lead to inadequate administration, also in case of tinea [19]. The risk of improper use of topical calcineurin inhibitors is lower because of their higher cost [19]. Statistical data from medical papers indicate that a majority of cases of tinea incognito have been previously treated by non-dermatologists or self-treated by patients [13].

Apart from mycological examination, another useful diagnostic tool, available mainly for dermatologists, is dermoscopy. According to the literature, the most commonly observed findings are: dotted vessels (however, the distribution can vary from peripheral, in most cases, to patchy), white scales with peripheral distribution, and the presence of a ‘moth-eaten’ scale with an outward-peeling direction of the scale, which seems to be the most specific feature [2]. Another imaging technique that has been described to be useful is in vivo reflectance confocal microscopy, although it is surely not widely available [20]. Molecular methods are perhaps more sensitive than microscopic examination in the detection of dermatophytes [6], but unfortunately, they are not widely available, and we also are not able to perform such tests in our department. The last possibility is taking a skin lesion sample for histopathological examination; however, it is the most invasive of the described methods. The microscopic picture may vary between patients and is often unspecific, but features that can suggest tinea are: neutrophils in the stratum corneum, compact orthokeratosis, and the presence of fungal hyphae between two zones of cornified cells (‘sandwich sign’) [21].

The second aspect of tinea incognito is its management when it has already occurred. Taking a patient’s history is the easiest and most helpful tool at the same time. Exactly as in the described case, patients usually state that they stayed in fitted clothing in hot conditions of high humidity, which they associate with the subsequent appearance of lesions [3]. Then, they report the administration of glucocorticoids—first, with slight improvement, and suddenly, with great deterioration and expansion of skin lesions. These are the data that can point right away to the correct diagnosis.

Another problem associated with tinea incognito is the fact the administration of glucocorticoids may result in their severe side effects, such as skin atrophy, stretch-marks, hypopigmentation or teleangiectasias [19].

Of note, oral antifungal agents, the most commonly used being terbinafine and azoles, may lead to several side effects, which should be kept in mind and monitored (the list of antifungal drugs available in our country is listed in Table S2 in the Supplementary Materials). As for the azoles, itraconazole is probably most frequently advised. It may cause intermittent liver enzymes’ activity to increase, nausea, vomiting, constipation or diarrhea, as well as rash or urticaria [22]. It has different interactions with other drugs, but none have been described for simultaneous use with perindopril on insulin, as in described case. As for terbinafine use, it may also lead to the symptoms as mentioned above, and a very rare, but characteristic, side effect is neutropenia or agranulocytosis [23]. It is advisable to perform laboratory tests before introduction of antifungal therapy and monitor them during the course of treatment. It is noteworthy that the mentioned antifungal agents interact with many other drugs; therefore, it is crucial for the doctor to analyze all the medications the patient already takes.

## 4. Conclusions

Tinea incognito is an exacerbated manifestation of dermatophyte infection after incorrect systemic or topical administration of glucocorticoids. It is a common problem, especially occurring in medical practices other than dermatological, first, because clinical picture of tinea may be challenging to diagnose and could be mistaken with other dermatoses, and second, because there is inferior access to direct mycological examination in outpatient care other than dermatology. To avoid tinea incognito, every doctor has to keep in mind the fact that tinea may be a great mimicker of other dermatoses, depending on the localization, should not prescribe medications without microscopic confirmation of tinea, and should refer patients for dermatological consultation in case of any doubt. Ideally, it would be helpful to increase the availability of mycological examination in general practice. In dermatological practices, other more sophisticated methods could be used for establishing the diagnosis.

## Data Availability

Not applicable.

## Supplementary Materials

The following supporting information can be downloaded at: https://www.mdpi.com/article/10.3390/jof8030312/s1, Table S1. The most common conditions that could be mistaken with tinea depending on the particular location; Table S2. The list of topical and oral antifungal drugs available in our country.
