# Supplementary material for: Tinea Incognito—A Great Physician Pitfall
Source: J Fungi (Basel). 2022 Mar 18;8(3):312. doi: 10.3390/jof8030312 (PMC8951265; doi:10.3390/jof8030312)
Supplement: Supplementary file 1 [file jof-08-00312-s001.zip › jof-1640684-supplementary.pdf]

SUPPLEMENTARY FILES

Table S1. The most common conditions that could be mistaken with tinea depending on the particular location.

Face tinea	Trunk tinea
eczema<br>demodecosis<br>seborrhoeic dermatitis<br>lupus erythematosus<br>rosacea<br>impetigo contagiosum	eczema<br>lupus erythematosus<br>psoriasis<br>drug eruptions<br>lichen planus<br>impetigo contagiosum
**Groin tinea**	**Limbs tinea**
eczema<br>erythrasma<br>psoriasis	eczema<br>psoriasis<br>drug eruptions<br>lichen planus<br>lesions associated with venous insufficiency

Table S2. The list of topical and oral antifungal drugs available in our country.

Topical drugs		Oral drugs	
**Name**	**Formulation**	**Name**	**Formulation**
terbinafine	cream, aerosole	terbinafine	tablets
clotrimazole	cream, intravaginal capsules, liquid	itraconazole	capsules
econazole	cream	fluconazole	capsules
fenticonazole	intravaginal capsules	voriconazole	tablets
izoconazole	cream	ketoconazole	tablets
ketoconazole	cream, shampoo		
miconazole	cream, powder, gel, intravaginal tablets, aerosole		
posaconazole	suspension		
cyclopiroxolamine	liquid, cream		
cyclopirox	nail lacquer		
amorolfine	nail lacquer		
